# 17β-Estradiol Inhibits Proliferation and Oxidative Stress in Vascular Smooth Muscle Cells by Upregulating BHLHE40 Expression

**DOI:** 10.3389/fcvm.2021.768662

**Published:** 2021-11-30

**Authors:** Dan-dan Feng, Bin Zheng, Jing Yu, Man-li Zhang, Ying Ma, Xiao Hao, Jin-kun Wen, Xin-hua Zhang

**Affiliations:** ^1^Ministry of Education of China, The Key Laboratory of Neural and Vascular Biology, Department of Biochemistry and Molecular Biology, Hebei Medical University, Shijiazhuang, China; ^2^The Second Department of Respiratory and Critical Care Medicine, The Second Hospital of Hebei Medical University, Shijiazhuang, China; ^3^Department of Critical Care Medicine, The Second Hospital of Hebei Medical University, Shijiazhuang, China; ^4^Department of Biochemistry and Molecular Biology, Binzhou Medical University, Yantai, China

**Keywords:** 17β-Estradiol, VSMCs, BHLHE40, proliferation, oxidative stress

## Abstract

**Background:** Intimal hyperplasia is a major complication of restenosis after angioplasty. The abnormal proliferation and oxidative stress of vascular smooth muscle cells (VSMCs) are the basic pathological feature of neointimal hyperplasia. 17β-Estradiol can inhibit VSMCs proliferation and inflammation. However, it is still unclear whether and how 17β-Estradiol affects intimal hyperplasia.

**Methods:** The neointima hyperplasia was observed by hematoxylin/eosin staining. The expression of PCNA, cyclin D1, NOX1, NOX4 and p47^phox^ in neointima hyperplasia tissues and VSMCs was determined by qRT-PCR and Western blotting. MTS assay, cell counting and EdU staining were performed to detect cells proliferation. The oxidative stress was assessed by ROS staining.

**Results:** 17β-Estradiol suppressed carotid artery ligation-induced intimal hyperplasia, which is accompanied by an increase of BHLHE40 level. Furthermore, loss- and gain-of-function experiments revealed that BHLHE40 knockdown promotes, whereas BHLHE40 overexpression inhibits TNF-α-induced VSMC proliferation and oxidative stress. 17β-Estradiol inhibited TNF-α-induced VSMC proliferation and oxidative stress by promoting BHLHE40 expression, thereby suppressing MAPK signaling pathways. In addition, enforcing the expression of BHLHE40 leads to amelioration of intimal hyperplasia.

**Conclusions:** Our study demonstrates that 17β-Estradiol inhibits proliferation and oxidative stress *in vivo* and *in vitro* by promotion of BHLHE40 expression.

## Introduction

Vascular smooth muscle cell (VSMC), which plays a crucial role in maintaining vascular structure and function, is mainly subsistence in the medial layer of the blood vessel wall (1). Nevertheless, abnormal VSMC proliferation, migration, inflammation or oxidative stress could lead to vascular remodeling, which contributes to the development of a series of vascular diseases, such as atherosclerosis, hypertension and post-angioplasty restenosis (2, 3). Tumor Necrosis Factor-α (TNF-α) is one of the cytokines which are involved in systemic inflammation. It is reported that TNF-α greatly induces VSMC proliferation and takes part in the formation of neointimal in response to vascular injury (4, 5). Therefore, inhibiting TNF-α signaling may be a useful method for preventing cardiovascular diseases.

Class E basic helix-loop-helix protein 40 (BHLHE40) has been proposed as a transcriptional repressor, which negatively regulates the activity of the clock genes (6). The BHLHE40 protein is widely expressed in a variety of human tissues. Researchers demonstrate that BHLHE40 is closely involved in many kinds of biological processes like cell proliferation, senescence, inflammation and oxidative stress (7–10). A previous study showed that BHLHE40 inhibits high glucose–induced calcification/senescence by directly binding to the promoter region of lncRNA-ES3 in HA-VSMC (11). In addition, multiple reports provide strong support for the association between BHLHE40 and oxidative stress (12). In myogenic cells, downregulation of BHLHE40 significantly reduces mitochondrial efficiency, resulting in the burst of ROS (13). Increased ROS production is integral to hypertension and atherosclerosis burden in mouse, rat and human arteries (14–16). However, whether BHLHE40 participates in the regulation of vascular remodeling is largely unknown. Here we explored the function of BHLHE40 in ligation injury-induced intimal hyperplasia, providing causative evidence that proliferation and oxidative stress were negatively regulated by BHLHE40 protein in VSMC.

17β-Estradiol (E2), an endogenous estrogen secreted by the ovaries of women, plays a vasoprotective role through regulating injury-induced chemokine expression and leukocyte infiltration (17). Previous studies have shown that E2 prevents the formation of atherosclerosis by inhibiting the proliferation and inflammation of VSMC (18). Besides, E2 contributes to reducing in-stent restenosis in porcine coronary injury models via suppressing smooth muscle cells proliferation and improving vascular re-endothelialization (19). Furthermore, it has been known that estrogen treatment can effectively increase the interaction of ERα with NF-κB p50, and reduce the interaction of KLF5 with NF-κB p50 induced by high glucose, thereby inhibiting inflammatory response in VSMC (20). It is therefore significant to gain mechanistic insights into how E2 and VSMC proliferation/oxidative stress are involved in vascular remodeling.

In this study, we identify that E2 exerts a protective effect on carotid artery ligation by regulating BHLHE40 expression. Additionally, we find that the up-expression of BHLHE40 in VSMC results in the suppression of MAPK signaling pathway. Taken together, our findings provide potential therapeutic targets for restenosis.

## Materials and Methods

### Animal Model and Treatment

Animal experiments were approved by the Institutional Animal Care and Use Committee of Hebei Medical University (approval ID: HebMU 20080026). Eight-week-old C57BL/6N male mice were purchased from Vital River Laboratory Animal Technology Co., Ltd., (Beijing, China). Animals were housed in a climatically controlled environment, on a 12 h light/dark cycle, with free access to water and standard food *ad libitum*.

The mice carotid artery ligation model applied has been described previously (21). Briefly, C57BL/6N male mice were anesthetized with 1.5% isoflurane. The left common carotid arteries were exposed and completely ligated with a 6–0 silk suture under the left carotid artery bifurcation to induce intima formation. The silk suture was passed below the exposed left carotid artery but not tightened as the control (*n* = 10). E2 (Sigma, 50-28-2, Purity ≥98%) (0.02 mg·kg^−1^·day^−1^) was infused through subcutaneous osmotic minipump (Alzet, Model 2004, USA) implantation 7 days before ligation injury and continuing for 14 days thereafter (*n* = 10). Ligated animals without E2 treatment received DMSO and corn oil at an equivalent amount (*n* = 10). The pcDNA3.1-BHLHE40 plasmids (*n* = 10) or pcDNA3.1-vehicle plasmids (*n* = 10) were diluted with Entranster™ solution (Engreen Biosystem, Beijing, China) and 10% glucose mixture (1:1 v/v) to 0.5 μg/μL *in vivo*. Then, added 10 μL aforesaid mixture into the 90 μL of 20% F-127 pluronic gel (Sigma, 9003-11-6) at 4°C for 2 h. Immediately after ligation, the exposed carotid artery adventitial surface was treated with 100 μL pluronic gel containing plasmids. At 14 days after surgery, all animals were anesthetized and perfused with cold PBS, and tissues were harvested for follow-up experiments.

### Hematoxylin and Eosin (HE) Staining

For morphometric analyses, the arteries were fixed with 4% paraformaldehyde and embedded in paraffin. Four μm cross-sections were cut from the proximal carotid ligation site and prepared for hematoxylin and eosin (HE) staining. For each section, six random non-contiguous microscopic fields were analyzed. The neointimal area and intima-to-media ratio were calculated using Image-Pro Plus Analyzer (version 5.1) software (Media Cybernetics, Silver Spring, MD) in a blinded manner.

### Cell Culture and Treatment

Mouse aortic vascular smooth muscle cell (mVSMC) (ATCC, No. CRL-2797™) were cultured in low-glucose Dulbecco's modified Eagle's medium (DMEM, Gibco Life Technologies, Rockville, MD) supplemented with 10% fetal bovine serum (GEMINI, USA) and 1 × Penicillin-Streptomycin-Glutamine (Gbico, USA), containing 100 units/mL of penicillin and 100 μg/mL of streptomycin, cultured at 37°C with 5% CO_2_ atmosphere. VSMCs were blocked by incubation in serum-deprived DMEM at 80–90% confluence or 24 h before stimulated with TNF-α or E2.

### Cell Transfection

siRNAs targeting mouse BHLHE40 (si-BHLHE40) and negative control (si-Ctrl) were designed and synthesized by GenePharma (Shanghai, China). The siRNA sequences used in our studies were as follows:

**Table:** 

**Name**	**Sequences 5^**′**^to 3^**′**^**
BHLHE40	Sense: GGAGAACGUGUCAGCACAATT
	Antisense:UUGUGCUGACACGUUCUCCTT
Control	Sense: UUCUCCGAACGUGUCACGUTT
	Antisense:ACGUGACACGUUCGGAGAATT

The expression plasmids of BHLHE40 (pcDNA3.1-BHLHE40) were created by the placement of mouse BHLHE40 CDS region of mRNA into the pcDNA3.1 vector. The siRNAs or plasmids were transiently transfected into VSMC with Lipofectamine 2000 (*Invitrogen*) according to the manufacturer's protocols.

### Cell Counting

The cell number was determined by Countess™ Automated Cell Counter (*Invitrogen*). After different treatment, VSMCs were digested, resuspended and blown into its individual tube. Ten μL of the cell suspension was mixed with 10 μL of Trypan blue, and counted by an *Invitrogen* Countess. Untreated cells were used for the baseline count. Each sample was counted three times, and the average value from triplicate experiments was measured.

### MTS Assay

Cell viability was determined using the MTS assay, as previously described (22). In brief, 1 × 10 ^4^ cells/well were seeded into 96-well plates with 5 replicates for each group, The next day, the cells were pretreated in 100 μL serum-free medium for 24 h and then incubated with appropriate treatment. The cells were incubated with CellTiter 96 AQueous One Solution (Promega, G3582) for 3 h, and the absorbance at 490 nm was measured using a Multiskan Spectrum (Thermo).

### Isolation of RNA and Real-Time PCR

Total RNA was extracted from VSMC or mouse aortic tissues using Trizol (*Invitrogen*™) according to the manufacturer's instruction. The concentration and purity of the extracted RNA were detected by NanoDrop ND-2000 spectrophotometer (Thermo Fisher, Waltham, USA).cDNA was synthesized using an M-MLV First Strand Kit (Life Technologies) and real-time PCR analysis was done with the BIO-RAD CFX96^TM^ Real-Time System, using the Platinum SYBR Green qPCR SuperMix UDG Kit (*Invitrogen*). Relative mRNA expression levels were normalized to 18S. All PCRs were performed in triplicate. Relative amount of transcripts was calculated using the 2^−Δ*ΔCt*^ formula.

The primer sequences were as follows:

**Table:** 

**Name**	**Sequences 5^**′**^to 3^**′**^**
18s	Forward: CGCCGCTAGAGGTGAAATTC
	Reverse: CCAGTCGGCATCGTTTATGG
PCNA	Forward: GGAGAGCTTGGCAATGGGAA
	Reverse: TAGGAGACAGTGGAGTGGC
cyclin D1	Forward: TGCCATCCATGCGGAAA
	Reverse: AGCGGGAAGAACTCCTCTTC
NOX1	Forward: GTGCCTTTGCCTGGTTCAACAAC
	Reverse: AGCCAGTGAGGAAGAGACGGTAG
NOX4	Forward: CTGGAAGAACCCAAGTTCCA
	Reverse: CTGATGCATCGGTAAAGTCT
p47^phox^	Forward: ATTCACCGAGATCTACGAGTTC
	Reverse: TGAAGTATTCAGTGAGAGTGCC
KLF4	Forward: CTAACCGTTGGCGTGAGGAACTC
	Reverse: TCTAGGTCCAGGAGGTCGTTGAAC
BHLHE40	Forward: GGAGAGGCGAGGTTACAGTG
	Reverse: AATGCCAGGCACATGACAAG

### Immunofluorescence Staining

Immunofluorescence staining was performed on 4 μm paraffin cross-sections from mouse artery samples. The sections were deparaffinized with xylene and rehydrated, and then were permeabilized by incubation with 0.5% Triton X-100 in phosphate-buffered saline (PBS). Non-specific sites were blocked by incubation in 10% normal goat serum (710027, KPL, USA) for 30 min. Then the sections were incubated with primary antibodies at 4°C overnight. The primary antibodies were mouse anti-SMα-actin (sc-130617, Santa Cruz) and rabbit anti-BHLHE40 (NB100-1800, Novus). Secondary antibodies were rhodamine-labeled antibody to rabbit IgG (031506, KPL, USA) and fluorescein-labeled antibody to mouse IgG (021815, KPL, USA). Nuclei were stained with DAPI (0100-20, SouthernBiotech) in each experiment. Images were captured by confocal microscopy (DM6000 CFS, Leica) and processed by LAS AF software.

### Immunohistochemistry

Immunohistochemical staining was visualized by use of an SPN-9001 Histostain^TM^-SP kit (Zhongshan Goldenbridge Biotechnology, Beijing, China) according to the manufacturer's instruction. Paraffin cross-sections were deparaffinized with xylene and rehydrated in a graded ethanol series, and endogenous peroxidase activity was inhibited by incubation with 3% H_2_O_2_ for 30 min. Sections were blocked with 10% normal goat serum for 10 min and incubated overnight at 4°C with anti-BHLHE40 antibody (1:100 dilution, NOVUS, NB100-1800). After a PBS wash, sections were incubated with secondary antibody at 37°C for 30 min. Drops of horseradish enzyme labeled streptomycin were added for 15 min, washed with PBS for 5 min and three times and then DAB staining was performed under the ordinary light microscope. Sections were counterstained with hematoxylin to visualize nuclei.

### ROS Assay

The intracellular ROS levels were measured following the instruction of Reactive Oxygen Species Assay Kit (Beyotime Biotechnology, China). Briefly, the cells were seeded in 12-well plates with microscope cover glasses and exposed to various treatments. The treated cells were loaded with 10 μM/L DCFH-DA at 37°C for 20 min. Subsequently, cells were washed with PBS three times and then observed using fluorescence microscopy (Olympus).

### Western Blot Analysis

Protein was isolated from VSMC or aortic tissues as the manufacturer's instruction of RIPA Lysis Buffer (Solarbio, Beijing, China). Equal amounts of protein were electrophoresed on 10% SDS-PAGE and transferred onto a PVDF membrane (Millipore). Membranes were blocked with 5% milk in TBS-Tween-20 (TBST) for 1.5 h at 37°C and incubated overnight at 4°C with the following primary antibodies: anti-PCNA (1:1000, ab92552, Abcam), anti-cyclin D1 (1:1000, 60186-1-Ig, Proteintech), anti-NOX1 (1:500, DF8684, Affinity Biotech), anti-NOX4 (1:500, 14347-1-AP, Proteintech), anti-p47^phox^ (1:1000, 4312, Cell Signaling Technology), anti-KLF4 (1:1000, GTX101509, GeneTex), anti-BHLHE40 (1:500, 17895-1-AP, Proteintech), anti-p44/42 MAPK (ERK1/2) (1:1000, 9102, Cell Signaling Technology), anti-phospho-p44/42 MAPK (ERK1/2) (Thr202/Tyr204) (1:1000, 4370, Cell Signaling Technology), anti-JNK (1:500, 9252, Cell Signaling Technology), anti-phospho-SAPK/JNK (Thr183/Tyr185) (1:500, 4668, Cell Signaling Technology), anti-p38 MAPK (1:500, 9212, Cell Signaling Technology), anti-phospho-p38 MAPK (Thr180/Tyr182) (1:500, 4511, Cell Signaling Technology) and anti-β-actin antibody (1:2000, ab6276, Abcam). Membranes were then incubated with secondary antibody (1:10000, Rockland) for 1.5 h at room temperature. At last, protein blots were treated with the Immobilon^TM^ western chemiluminescent HRP substrate (Millipore) and detected by ECL (enhanced chemiluminescence) Fusion Fx (Vilber Lourmat). Images were captured and processed by FusionCapt Advance Fx5 software (Vilber Lourmat).

### EdU Incorporation Assay

The EdU incorporation assay was carried out according to the manufacturer's instruction (RiboBio, China). The representative images acquired by fluorescence microscope (Olympus). The cell proliferative rate was calculated as the proportion of Hoechst 33342-staining cells that incorporated EdU in 10 high-power fields per well.

### Statistical Analysis

Data are expressed as the means ± S.E.M. of at least three independent experiments. All analyses were performed using GraphPad Prism 5.0 software (GraphPad Software, La Jolla, CA). Differences between two groups were analyzed by Student's *t*-test. For multiple comparisons or repeated measurements, ANOVA or repeated ANOVA followed by a Tukey's *post-hoc* test was used. A value of *p* < 0.05 was considered statistically significant.

## Results

### E2 Significantly Decreases Neointimal Formation, Proliferation and Oxidative Stress Induced by Carotid Artery Ligation

**Figure 1:**
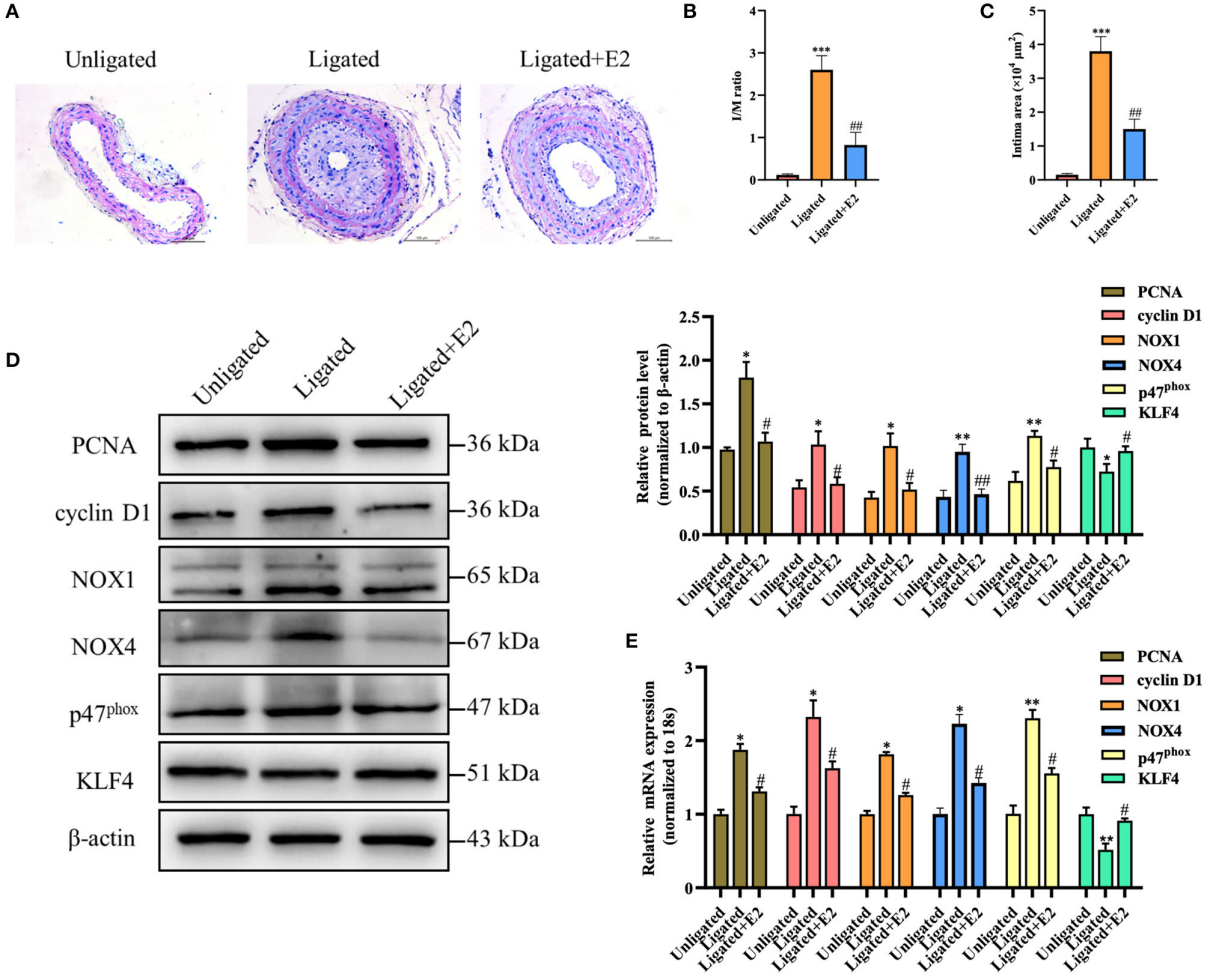
E2 attenuates neointimal formation, proliferation and oxidative stress induced by carotid artery ligation. **(A)** Representative photomicrographs of the HE-stained sections of carotid arteries from unligated vessels, ligated vessels, and ligated vessels treated with E2 (*n* = 4). Scale bars = 100 μm. **(B,C)** Morphometric quantification of I/M ratio and the intima area in the different groups. ***p < 0.001 vs. Unligated group, ^##^*p* < 0.01 vs. Ligated group. **(D)** PCNA, cyclin D1, NOX1, NOX4, p47^phox^ and KLF4 expression in unligated, ligated and ligated + E2-treated carotid arteries was detected by Western blotting. Statistic of band intensities is shown on the right (*n* = 3). *p < 0.05 and **p < 0.01 vs. Unligated group, ^#^*p* < 0.05 and ^*##*^*p* < 0.01 vs. Ligated group. **(E)** PCNA, cyclin D1, NOX1, NOX4, p47^phox^ and KLF4 expression in unligated, ligated and ligated + E2-treated carotid arteries was detected by qRT-PCR (*n* = 3). *p < 0.05 and **p < 0.01 vs. Unligated group, ^#^*p* < 0.05 vs. Ligated group.

HE staining showed that carotid arterial intima thickness was significantly increased in ligation injury-induced intimal hyperplasia mice models at 14 days post-operation. Compared with the ligated group, the degree of neointimal formation was obviously reduced in E2-treated group (Figure 1A). The ratio of intima to media (I/M ratio) and intimal area were dramatically lower in E2-treated group than that in the ligated group (Figures 1B,C). These results indicate that E2 can effectively inhibit neointimal formation induced by carotid artery ligation. Since it is known that ligation injury-induced intimal hyperplasia is closely related to VSMC proliferation and oxidative stress, we next investigate the effects of E2 on proliferation and oxidative stress-related genes expression in carotid arteries. Western blotting analysis revealed that vascular injury increased the expression of PCNA, cyclin D1, NOX1, NOX4 and p47^phox^, whereas KLF4 expression was remarkably downregulated. Notably, carotid artery ligation-induced these changes were reversed by E2 (10 mg ·kg^−1^·day^−1^) treatment (Figure 1D). qRT-PCR analysis of PCNA, cyclin D1, NOX1, NOX4, p47^phox^ and KLF4 expression was consistent with their expression of protein level (Figure 1E). Overall, these studies demonstrated E2 could alleviate vascular remodeling in intimal hyperplasia mice partly through limiting the proliferation and oxidative stress of VSMC.

### E2 Inhibits TNF-α-Induced VSMC Proliferation and Oxidative Stress

**Figure 2:**
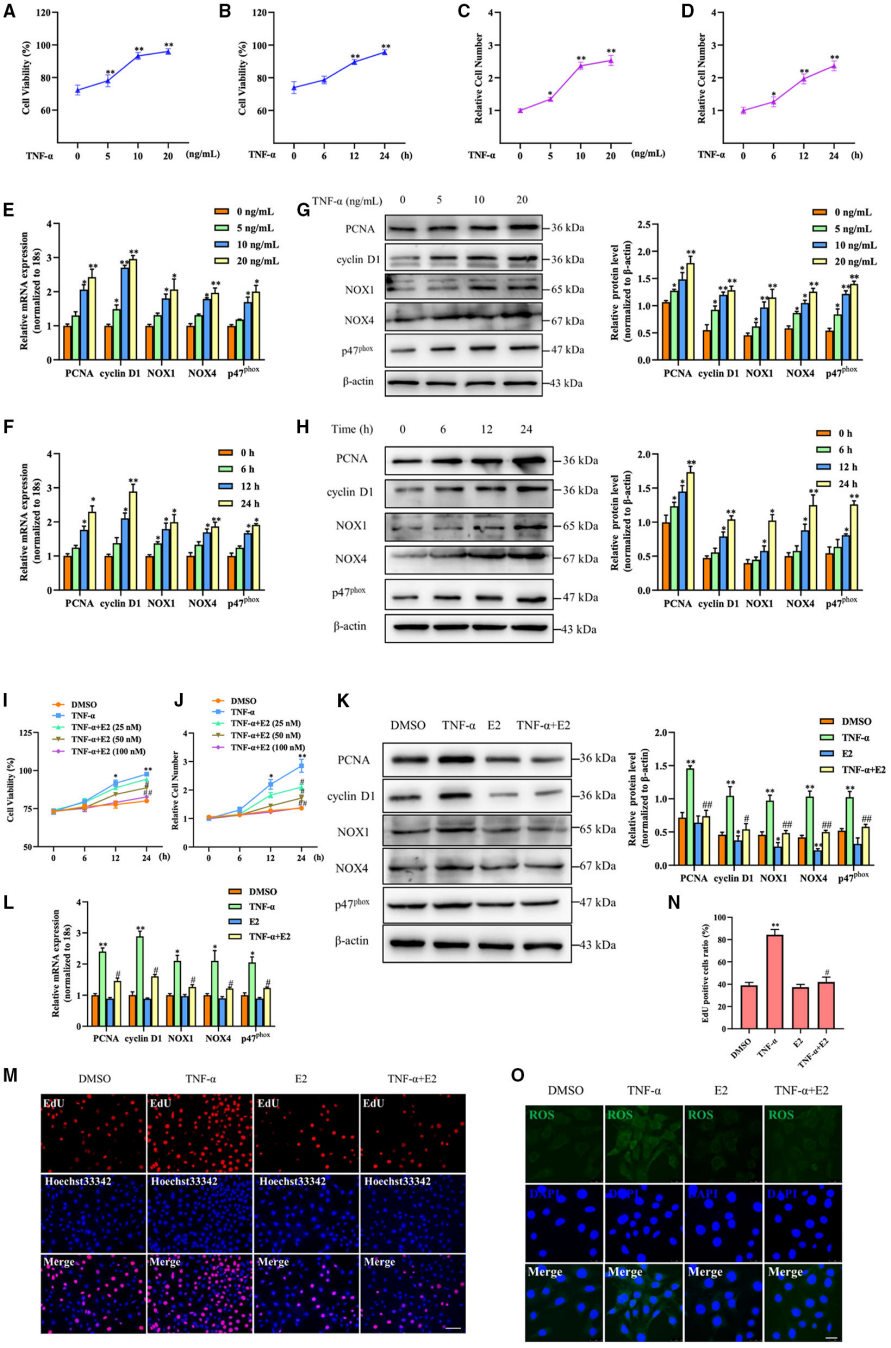
E2 inhibits TNF-α-induced proliferation and oxidative stress in VSMC. **(A–H)** VSMCs were stimulated with TNF-α for indicated doses and times. The cell viability was determined by MTS assay **(A,B)**. *p < 0.05 and **p < 0.01 vs. untreated group, respectively. Cell counting was carried out using a Countess automated counter **(C,D)**. *p < 0.05 and **p < 0.01 vs. untreated group, respectively. qRT-PCR detected the mRNA expression of PCNA, cyclin D1, NOX1, NOX4 and p47^phox^ **(E,F)**. *p < 0.05 and **p < 0.01 vs. untreated group, respectively. Western blotting detected PCNA, cyclin D1, NOX1, NOX4 and p47^phox^ protein expression **(G,H)**. Statistic of band intensities is shown on the right. *p < 0.05 and **p < 0.01 vs. untreated group, respectively. **(I,J)** VSMCs were pretreated with 25, 50 and 100 nM of E2 for 6 h and then were stimulated with TNF-α (10 ng/mL) for the indicated times. The cell viability was determined by MTS assay **(I)**, and cell counting was carried out using a Countess automated counter **(J)**. *p < 0.05 and **p < 0.01 vs. DMSO group, ^#^*p* < 0.05 and ^##^ *p* < 0.01 vs. TNF-α group. **(K–O)** VSMCs were pretreated with E2 (100 nM) for 6 h and then were stimulated with TNF-α (10 ng/mL) for 24 h. PCNA, cyclin D1, NOX1, NOX4 and p47^phox^ expression was determined by Western blotting **(K)** and qRT-PCR **(L)**. Statistic of band intensities is shown on the right. *p < 0.05 and **p < 0.01 vs. DMSO group, ^#^*p* < 0.05 and ^##^*p* < 0.01 vs. TNF-α group. Cell proliferation was detected by EdU staining **(M)**. Scale bar = 100 μm. Analysis of the percentage of EdU positive cells **(N)**. **p < 0.01 vs. DMSO group, ^#^*p* < 0.05 vs. TNF-α group. ROS levels were detected by DCFH-DA staining **(O)**. Scale bar = 25 μm.

Because it is known that TNF-α stimulates VSMC proliferation and oxidative stress, we sought to determine whether E2 suppressed neointimal hyperplasia through restraining TNF-α-induced VSMC proliferation and oxidative stress. As shown in Figures 2A–D, TNF-α treatment markedly increased VSMC viability and number in a dose and time-dependent manner by MTS assay and cell counting. Simultaneously, exposure of VSMC to TNF-α dose and time-dependently enhanced mRNA and protein expression of PCNA, cyclin D1, NOX1, NOX4 and p47^phox^ (Figures 2E–H). Next, we detected the effects of E2 treatment on VSMC proliferation and oxidative stress induced by TNF-α. As shown by MTS assay and cell counting, treating VSMC with TNF-α (10 ng/mL) promoted cell proliferation in a time-dependent manner, whereas pretreatment of VSMC with 25, 50 and 100 nM of E2 for 6 h dose-dependently abrogated the inducing effects of TNF-α on VSMC viability and number (Figures 2I,J). Western blotting and qRT-PCR assay displayed that E2 offsets the up-regulation of PCNA, cyclinD1, NOX1, NOX4 and p47^phox^ expression induced by TNF-α (Figures 2K,L). In addition, EdU staining proved that E2 reversed TNF-α-induced VSMC proliferation (Figures 2M,N). In Figure 2O, E2 also visibly blocked TNF-α-induced ROS production in VSMC. In general, these results indicate that E2 inhibits TNF-α-induced VSMC proliferation and oxidative stress.

### E2 Promotes the Expression of BHLHE40 Both *in vivo* and *in vitro*

**Figure 3:**
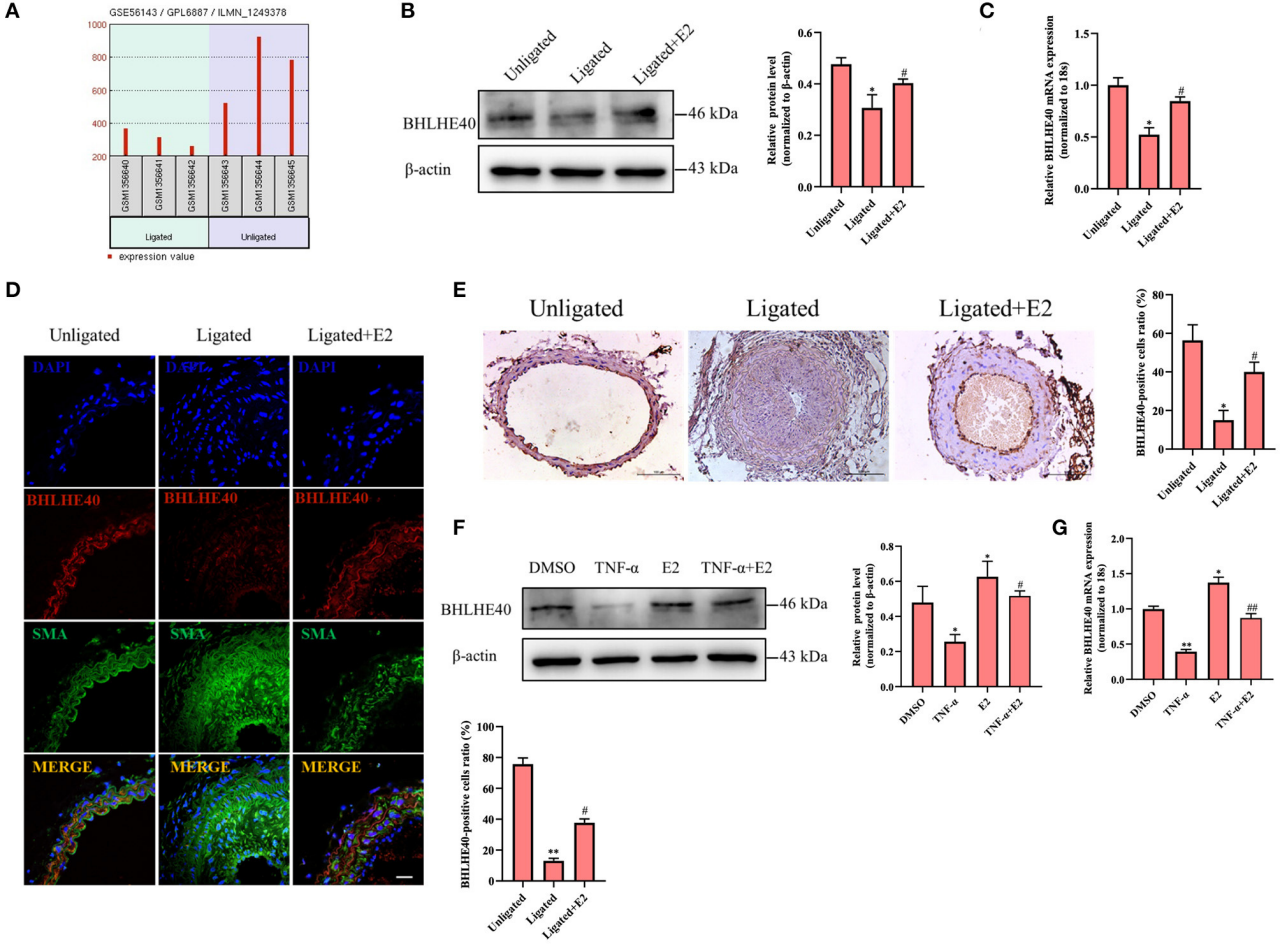
E2 promotes the expression of BHLHE40 both *in vivo* and *in vitro*. **(A)** The data of BHLHE40 expression was downloaded from the GEO databases (GSE56143). **(B,C)** BHLHE40 expression in unligated, ligated and ligated + E2-treated carotid arteries was detected by Western blotting **(B)** and qRT-PCR **(C)**. Statistic of band intensities is shown on the right. *p < 0.05 vs. Unligated group, ^#^*p* < 0.05 vs. Ligated group. **(D)** Immunofluorescence staining of a-SMA (SMA; green), BHLHE40 (red) and the nucleus (DAPI; blue) in unligated, ligated and ligated + E2-treated carotid arteries. Scale bars = 25 μm. Statistics of BHLHE40-positive cells unligated, ligated and ligated + E2-treated carotid arteries is shown on the right. **p < 0.01 vs. Unligated group, ^#^*p* < 0.05 vs. Ligated group. **(E)** Immunochemistry staining of BHLHE40 in unligated, ligated and ligated + E2-treated carotid arteries. Scale bars = 100 μm. Statistics of BHLHE40-positive cells unligated, ligated and ligated + E2-treated carotid arteries is shown on the right. *p < 0.05 vs. Unligated group, ^#^*p* < 0.05 vs. Ligated group. **(F,G)** VSMCs were pretreated with 100 nM of E2 for 6 h and then were stimulated with TNF-α (10 ng/mL) for 24 h, the expression of BHLHE40 was determined by Western blotting **(F)** and qRT-PCR **(G)**. Statistic of band intensities is shown on the right. *p < 0.05 and **p < 0.01 vs. DMSO group, ^#^*p* < 0.05 and ^##^*p* < 0.01 vs. TNF-α group.

In order to obtain which genes have been changed during neointimal hyperplasia, we downloaded an expression dataset (GSE56143) from the Gene Expression Omnibus (GEO), and found that the rhythm gene BHLHE40 was down-regulated in the ligated vascular tissue (Figure 3A). It has been reported that BHLHE40 can participate in the occurrence and development of cancer (23), but its role in the regulation of proliferation and oxidative stress in VSMC is still unclear. Therefore, we focused our research on BHLHE40. Western blotting and qRT-PCR assay showed that compared with unligated tissues, protein and mRNA expression levels of BHLHE40 were down-regulated by more than 0.5 times at 14 days after carotid artery ligation (Figures 3B,C). Furthermore, both immunofluorescence staining and immunochemistry staining of BHLHE40 were markedly reduced in injured arteries compared to sham-operation. Noticeably, carotid artery ligation-induced downregulation of BHLHE40 was reversed by E2 (Figures 3D,E). Western blotting (Figure 3F) and qRT-PCR assay (Figure 3G) revealed that TNF-α treatment lessened protein and mRNA expression of BHLHE40 compared with the control group, whereas pretreatment with E2 (100 nM) largely counteracted the inhibitory effects of TNF-α on BHLHE40 expression. Taken together, these findings suggest that E2 promotes the expression of BHLHE40 both *in vivo* and *in vitro*.

### Knockdown of BHLHE40 Promoted TNF-α-Induced VSMC Proliferation and Oxidative Stress

**Figure 4:**
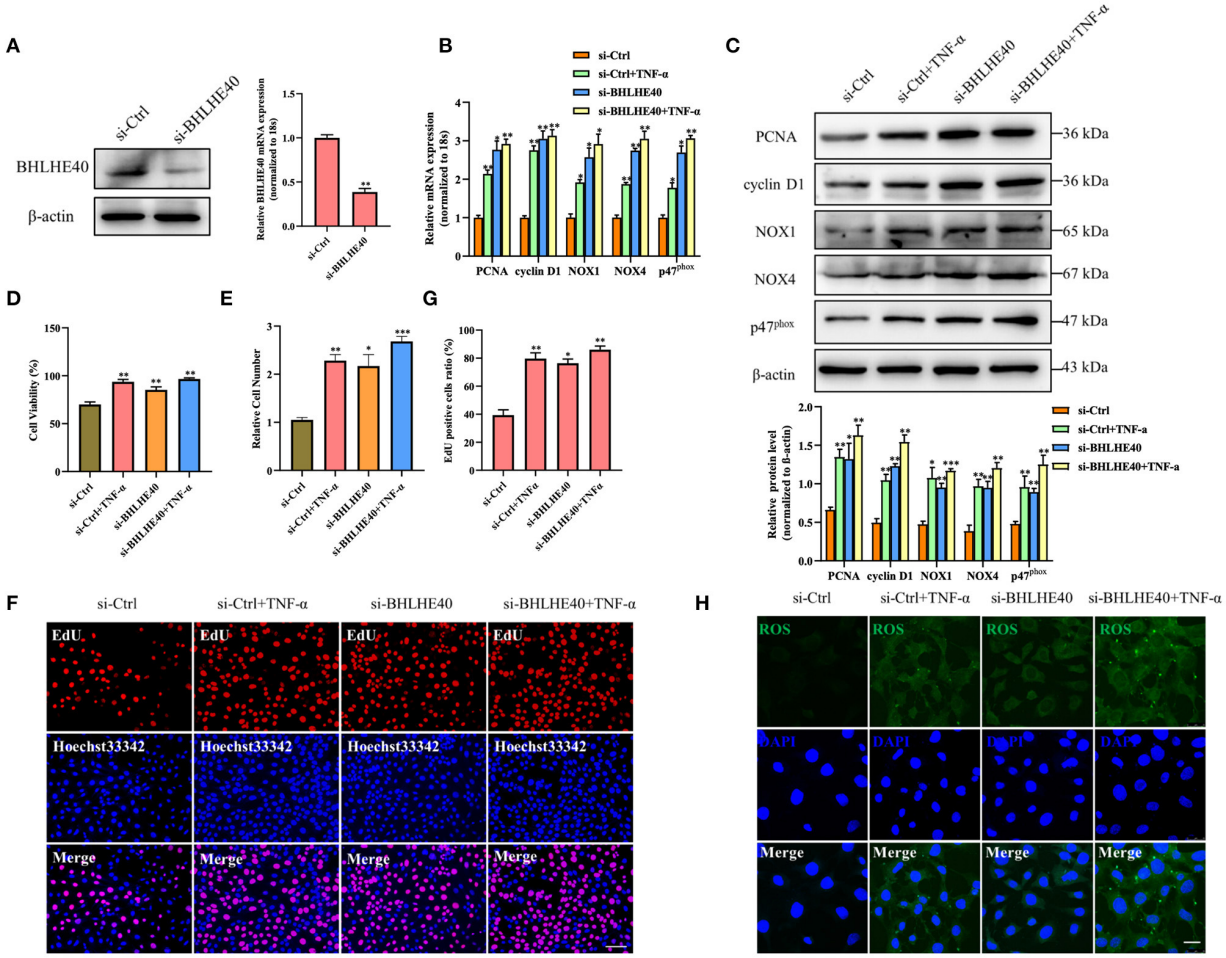
Downregulation of BHLHE40 promotes TNF-α-induced proliferation and oxidative stress in VSMC. **(A)** VSMCs were transfected with si-BHLHE40, and the expression of BHLHE40 was detected by Western blotting and qRT-PCR. **p < 0.01 vs. si-Ctrl group. **(B–H)** VSMCs were transfected with si-BHLHE40 and then treated or not with TNF-α for 24 h. The expression of PCNA, cyclin D1, NOX1, NOX4 and p47^phox^ was determined by qRT-PCR **(B)** and Western blotting **(C)**. Statistic of band intensities is shown on the bottom. *p < 0.05, **p < 0.01 and ***p < 0.001 vs. si-Ctrl group. The cell viability was determined by MTS assay **(D)**, and cell counting was carried out using a Countess automated counter **(E)**. *p < 0.05, **p < 0.01 and ***p < 0.001 vs. si-Ctrl group. Cell proliferation was detected by EdU staining **(F)**. Scale bar = 100 μm. Analysis of the percentage of EdU positive cells **(G)**. *p < 0.05, and ** *p* < 0.01 vs. si-Ctrl group. ROS levels were detected by DCFH-DA staining **(H)**. Scale bar = 25 μm.

To further illustrate the role of BHLHE40 in ligation injury-induced intimal hyperplasia, we assayed the effects of BHLHE40 down-regulation on cellular proliferation and oxidative stress in VSMC. Firstly, we confirmed that the expression of BHLHE40 at the protein and mRNA levels was silenced by about 70% in si-BHLHE40 transfected VSMC (Figure 4A). Subsequently, we examined the effects of si-BHLHE40 on the expression of proliferation and oxidative stress-related genes, and found that treating VSMC with TNF-α clearly increased the expression of PCNA, cyclinD1, NOX1, NOX4 and p47^phox^, which was enforced by si-BHLHE40 transfection (Figures 4B,C). In follow-up experiments, we found that BHLHE40 knockdown increased TNF-α-induced proliferation in VSMC, as shown by MTS analysis and cell counting (Figures 4D,E). Meanwhile, EdU staining evidenced that depletion of BHLHE40 by its siRNA increased TNF-α-induced VSMC proliferation (Figures 4F,G). In Figure 4H, ROS staining showed that si-BHLHE40 and TNF-α co-treatment further enhanced TNF-α-induced ROS production in VSMC. All in all, these data suggested that knockdown of BHLHE40 contributes to TNF-α-induced VSMC proliferation and oxidative stress.

### Overexpression of BHLHE40 in VSMC Inhibits Cell Proliferation and Oxidative Stress

**Figure 5:**
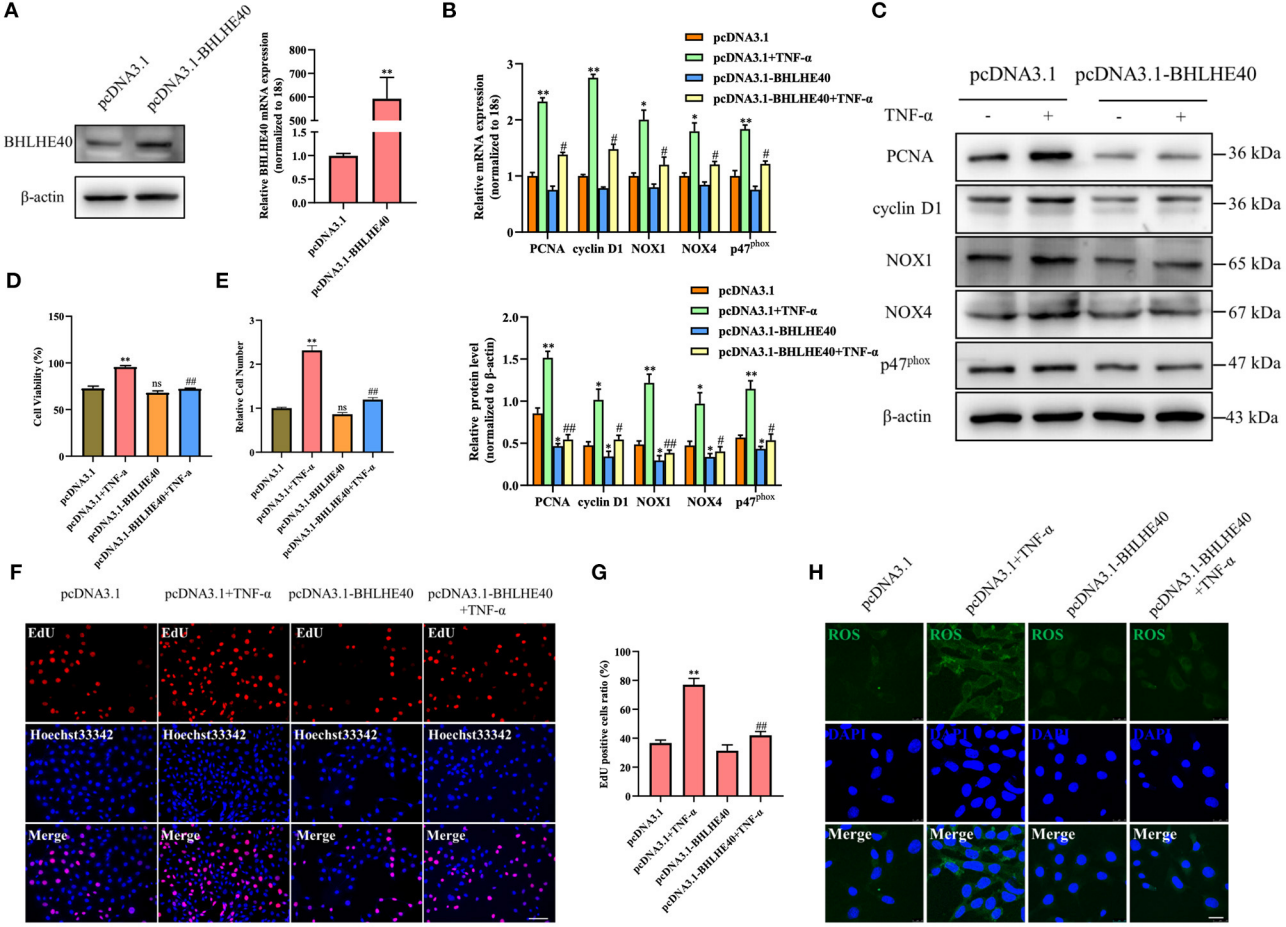
Overexpression of BHLHE40 inhibits TNF-α-induced proliferation and oxidative stress in VSMC. **(A)** VSMCs were transfected with pcDNA3.1-BHLHE40, and the expression of BHLHE40 was detected by Western blotting and qRT-PCR. **p < 0.01 vs. pcDNA3.1 group. **(B–H)** VSMCs were transfected with pcDNA3.1-BHLHE40 and then treated or not with TNF-α for 24 h. The expression of PCNA, cyclin D1, NOX1, NOX4 and p47^phox^ was determined by qRT-PCR **(B)** and Western blotting **(C)**. Statistic of band intensities is shown on the left. *p < 0.05 and **p < 0.01 vs. pcDNA3.1 group, ^#^*p* < 0.05 and ^##^*p* < 0.01 vs. pcDNA3.1 + TNF-α group. The cell viability was determined by MTS assay **(D)**, and cell counting was carried out using a Countess automated counter **(E)**. **p < 0.01 vs. pcDNA3.1 group, ^##^*p* < 0.01 vs. pcDNA3.1 + TNF-α group. Cell proliferation was detected by EdU staining **(F)**. Scale bar = 100 μm. Analysis of the percentage of EdU positive cells **(G)**. **p < 0.01 vs. pcDNA3.1 group, ^##^*p* < 0.01 vs. pcDNA3.1 + TNF-α group. ROS levels were detected by DCFH-DA staining **(H)**. Scale bar = 25 μm.

Next, we successfully overexpressed the BHLHE40 at both mRNA and protein level in VSMC (Figure 5A). To further explore whether BHLHE40 participates in the induction of proliferation and oxidative stress in TNF-α-treated VSMC, we forcedly expressed BHLHE40 and found that BHLHE40 overexpression distinctly reduced the expression of PCNA, cyclinD1, NOX1, NOX4 and p47^phox^ induced by TNF-α at both mRNA and protein levels (Figures 5B,C). As presented by MTS assay and cell counting, overexpression of BHLHE40 efficaciously counteracted the stimulatory effect of TNF-α on VSMC proliferation (Figures 5D,E). Similarly, EdU staining showed that the enforced expression of BHLHE40 in VSMC had opposite effects on TNF-α-induced proliferation (Figures 5F,G). Up-regulation of BHLHE40 led to a decrease in the production of TNF-α-induced ROS (Figure 5H). Altogether, these results indicate that BHLHE40 negatively regulates the proliferation and oxidative stress of VSMC by affecting the expression of PCNA, cyclinD1, NOX1, NOX4 and p47^phox^.

### BHLHE40 Suppressed Proliferation and Oxidative Stress Responses Through Inhibiting MAPK Signaling Pathway

**Figure 6:**
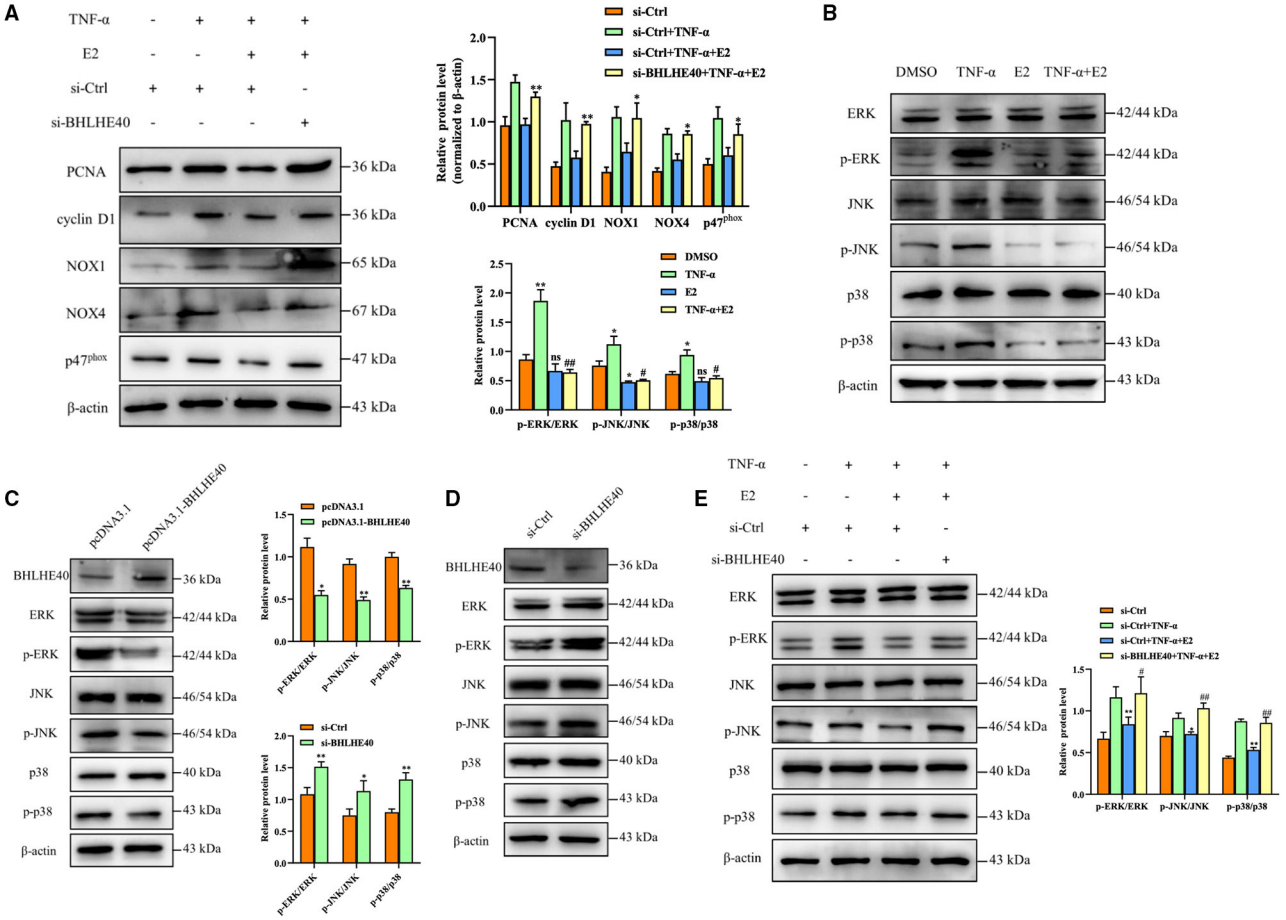
BHLHE40 suppresses proliferation and oxidative stress responses through suppressing MAPK signaling pathways. **(A)** VSMCs were transfected with si-BHLHE40 or si-Ctrl and then treated with the indicated treatments. The expression of PCNA, cyclin D1, NOX1, NOX4 and p47^phox^ was determined by Western blotting. Statistic of band intensities is shown on the right. *p < 0.05 and **p < 0.01 vs. si-Ctrl + TNF-α + E2 group. **(B)** VSMCs were pretreated with E2 (100 nM) for 6 h and then were stimulated with TNF-α (10 ng/mL) for 24 h. Western blotting was performed for total and phosphorylated ERK, JNK and p38. Statistic of band intensities is shown on the left. *p < 0.05 and ** *p* < 0.01 vs. DMSO group, ^#^*p* < 0.05 and ^##^*p* < 0.01 vs. TNF-α group. **(C)** VSMCs were transfected with pcDNA3.1- BHLHE40 for 24 h, and Western blotting analysis was performed for total and phosphorylated ERK1/2, JNK and p38. Statistic of band intensities is shown on the right. *p < 0.05 and **p < 0.01 vs. pcDNA3.1 group. **(D)** VSMCs were transfected with si-BHLHE40 for 24 h, and Western blotting analysis was performed for total and phosphorylated ERK, JNK and p38. Statistic of band intensities is shown on the left. *p < 0.05 and **p < 0.01 vs. si-Ctrl group. **(E)** VSMCs were transfected with si-BHLHE40 or si-Ctrl and then treated with the indicated treatments. Total protein lysates were collected and the expression of ERK, JNK and p38 and their phosphorylated forms were examined by Western blotting. Statistic of band intensities is shown on the right. *p < 0.05 and **p < 0.01 vs. si-Ctrl + TNF-α, ^#^*p* < 0.05 and ^##^*p* < 0.01 vs. si-Ctrl + TNF-α + E2 group.

Next, we performed BHLHE40 knockdown experiment to investigate whether BHLHE40 mediates the inhibitory role of E2 in the proliferation and oxidative stress of VSMCs. As shown in Figure 6A, down-regulation of BHLHE40 can reverse the inhibitory effects of E2 on the proliferation and oxidative stress. It is known that MAPK cascade activation is the center of multiple signaling pathways, and plays a key role in cell proliferation, inflammation and oxidative stress. Western blotting analysis revealed that TNF-α treatment markedly increased phosphorylation of ERK, JNK and P38 in VSMC, but the effects of TNF-α on MAPK signaling pathways were normalized by E2 treatment (Figure 6B). In order to clarify the mechanism by which BHLHE40 regulates proliferation and oxidative stress, we up-regulated or down-regulated the expression of BHLHE40 in VSMC, and monitored the expression of related genes in the MAPK signaling pathway. As shown in Figure 6C, up-regulation of BHLHE40 can lead to decreased ERK, JNK and p38 phosphorylation. On the contrary, down-regulating the expression of BHLHE40 can usefully increase ERK, JNK and p38 phosphorylation (Figure 6D). In order to confirm whether E2 regulates the MAPK signaling pathway by affecting the expression of BHLHE40, we conducted rescue experiments. As demonstrated in Figure 6E, TNF-α-induced phosphorylation of ERK, JNK and P38 were partly inhibited after E2 preincubation (Figure 6E, lane 3 vs. lane 2). Knockdown of BHLHE40 restrained this inhibitory effect of E2 (Figure 6E, lane 4 vs. lane 3). In addition, we examined the effect of E2 and BHLHE40 on AKT phosphorylation, as shown in Supplementary Figure S1, E2 treatment can lead to decreased AKT phosphorylation, but down-regulated BHLHE40 have no influence on the inhibitory effect of E2. On balance, the above results confirmed that 100 μM E2 displays suppressive effects on TNF-α-induced pathologic changes through deactivating MAPK signal pathways.

### BHLHE40 Overexpression Alleviated Neointimal Formation Induced by Carotid Artery Ligation Through Repressing Proliferation and Oxidative Stress in Arterial Walls

**Figure 7:**
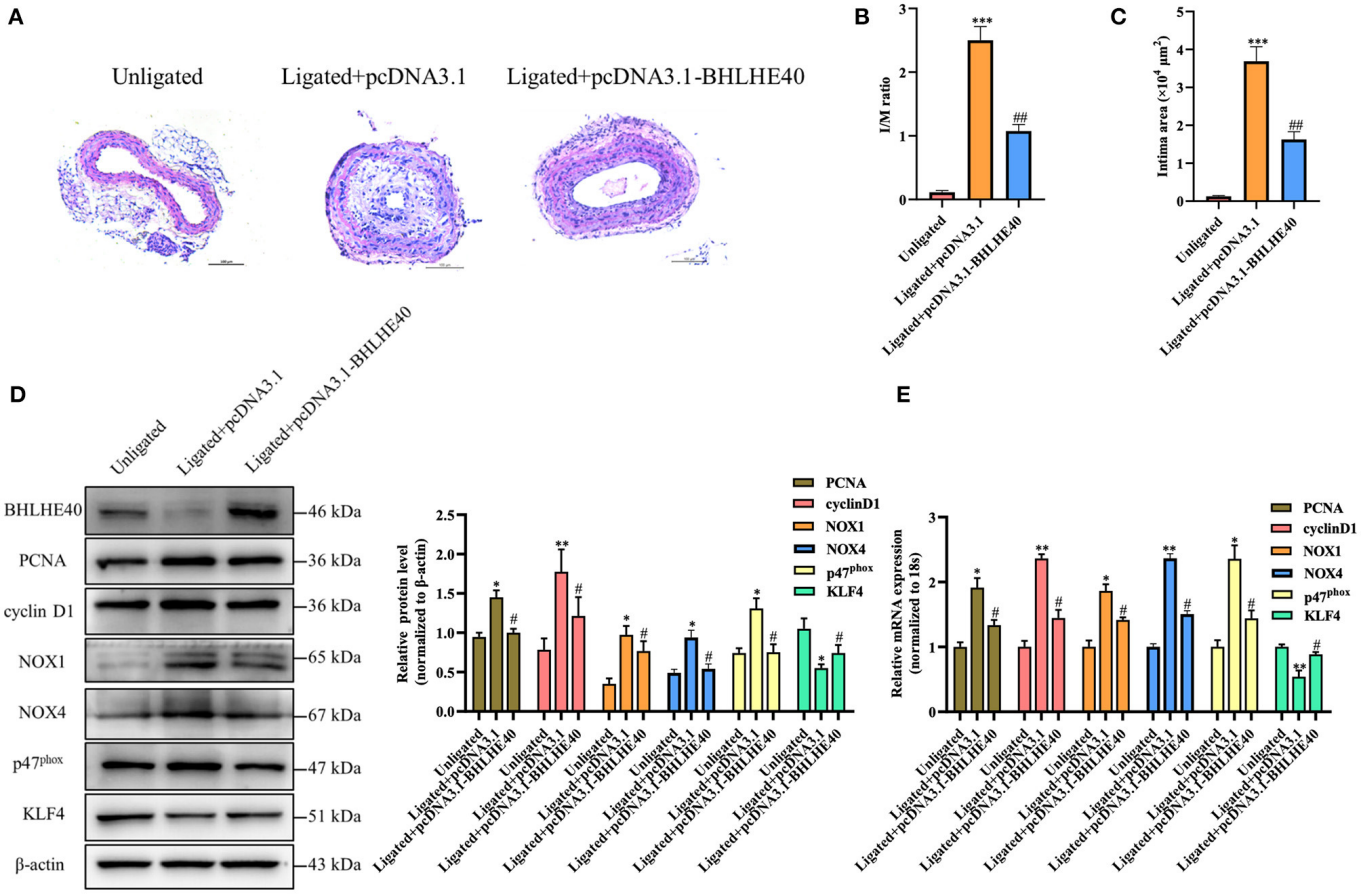
BHLHE40 overexpression alleviates neointimal formation, proliferation and oxidative stress induced by carotid artery ligation. **(A)** Representative photomicrographs of the HE-stained sections of carotid arteries from unligated vessels, ligated + pcDNA3.1 vessels, and ligated + pcDNA3.1-BHLHE40 vessels (*n* = 4). Scale bars = 100 μm. **(B,C)** Morphometric quantification of I/M ratio and the intima area in the different groups. ***p < 0.001 vs. Unligated group, ^##^*p* < 0.01 vs. Ligated + pcDNA3.1 group. **(D)** BHLHE40, PCNA, cyclin D1, NOX1, NOX4, p47^phox^ and KLF4 expression in unligated, ligated + pcDNA3.1 and ligated + pcDNA3.1-BHLHE40 carotid arteries was detected by Western blotting. Statistic of band intensities is shown on the right (*n* = 3). *p < 0.05 and **p < 0.01 vs. Unligated group, ^#^*p* < 0.05 vs. Ligated + pcDNA3.1 group. **(E)** PCNA, cyclin D1, NOX1, NOX4, p47^phox^ and KLF4 expression in unligated, ligated + pcDNA3.1 and ligated + pcDNA3.1-BHLHE40 carotid arteries was detected by qRT-PCR (*n* = 3). *p < 0.05 and **p < 0.01 vs. Unligated group, ^#^*p* < 0.05 vs. Ligated + pcDNA3.1 group.

To examine whether BHLHE40 is a key mediator in vascular remodeling, Pluronic F-127 gel solution containing pcDNA3.1 plasmids or pcDNA3.1-BHLHE40 plasmids were applied to the exposed adventitial surface of an ~5 mm segment of the ligated carotid artery. The intimal thickness of the ligated artery was determined 14 days after the surgery. As expected, carotid arterial ligation increased vascular wall thickness in control-plasmids transfected mice, and this expansion was strongly reduced in BHLHE40-plasmids transfected mice (Figure 7A). Consistent with these results, BHLHE40-overexpressed mice showed an important decrease in the ratio of intimal/medial area (I/M ratio) and neointimal area compared with control-plasmids transfected mice (Figures 7B,C). Next, we examined the expression of PCNA, cyclinD1, NOX1, NOX4 and p47^phox^ and KLF4 in the injured carotid artery of pcDNA3.1 plasmids or pcDNA3.1-BHLHE40 plasmids transfected mice. Notably, western blotting and qRT-PCR analysis data showed that carotid artery ligation-induced above gene changes were normalized by BHLHE40 overexpression (Figures 7D,E). To sum up, these data support the pathophysiological role of BHLHE40 depletion in vascular hypertrophy.

## Discussion

Vascular remodeling is the pathological basis of many cardiovascular diseases such as hypertension and atherosclerosis. The abnormal proliferation and oxidative stress of VSMC play an important role in the occurrence and development of vascular remodeling (24, 25). Evidence is also emerging to suggest that treatment of proliferation and oxidative stress of VSMC causes a reduction or prevents the progression of the carotid intima-media thickness, paralleled by a decrease in cardiovascular risk and events (26, 27). Therefore, exploring an effective treatment strategy to block the proliferation of VSMC and the occurrence of oxidative stress is essential for the treatment of cardiovascular diseases.

In this study, we showed that (1) E2 inhibited carotid artery ligation-induced intimal hyperplasia *in vivo* and TNF-α-induced VSMC proliferation and oxidative stress *in vitro*. (2) E2 inhibited TNF-α-induced VSMC proliferation and oxidative stress by increasing BHLHE40 expression, (3) Overexpression of BHLHE40 abolished TNF-α-induced VSMC proliferation and oxidative stress, (4) BHLHE40 mediated E2-induced suppression of MAPK signaling pathway expression, and (5) BHLHE40 overexpression protected against neointimal hyperplasia induced by carotid artery ligation.

17β-estrogen is a powerful steroid hormone, high in women from puberty to menopause and low in men. Anecdotal evidence suggested that the incidence of atherosclerosis in pre-menopausal women is much lower than that of age-matched males, but the discrepancy narrowed after post-menopausal in women, suggesting the preventive effect of estrogen on cardiovascular diseases (28, 29). Previous studies have indicated that 17β-estradiol treatment reduces neointimal hyperplasia and ameliorates re-endothelialization in injured carotid arteries (19, 30). It is well-known that a key mechanism for inhibiting intimal thickening is the repression of cell proliferation and oxidative stress (31, 32).In line with previous results, and our animal experiment data showing E2 can effectively improve neointimal hyperplasia in ligated carotid arteries by diminution of proliferation-related genes expression and attenuation of NADPH oxidase activity in VSMC. It has long been known that increased PCNA and cyclin D1 expression and enhanced ROS levels in VSMC exposed to TNF-α (33, 34). Our data showing E2 markedly inhibited the TNF-α-induced expression of PCNA, cyclin D1, NOX1, NOX4 and p47^phox^.

It is well-established that the MAPK signaling pathway regulates cellular proliferation, calcification, inflammation and oxidative stress (35, 36). Recently reports showed that increased phosphorylation of ERK1/2 expression contributes to the proliferation of VSMC (37, 38), Beyond cell proliferation, ERK 1/2 phosphorylation modulates VSMC phenotypic switch in Abdominal Aortic Aneurysms (39). In addition, p38 MAPK kinase promotes vascular calcification by inducing the expression of RUNX2 in VSMC (40). In primary mouse VSMC, p38 kinase is key to TGF-β-mediated growth inhibition (41). Previous studies showed that corylin treatment effectively attenuated atherosclerotic lesions by suppressing ROS production, VSMC proliferation and JNK phosphorylation in ApoE-deficient mice (42). Similarly, Ox-LDL induced oxidative stress promoted VSMC transformation from contraction to secretion via the JNK and ERK signaling pathways (43). Our recent study indicated that the activation of MAPK family members, such as ERK1/2, JNK and p38, was largely significantly abolished by E2 in TNF-α-induced VSMC.

Recently, an increasing number of reports have clarified the regulatory mechanisms mediated by BHLHE40 and its associations with the etiopathogenesis of various diseases (44, 45). For example, BHLHE40 directly interacts with estrogen receptor α to suppress the proliferation of ER-positive breast cancer cells (46). According to the newest reports, BHLHE40 deficiency resulted in accelerated osteopenia through attenuated PI3KCA/Akt/GSK3β signaling (47). In addition, the high expression of BHLHE40 in gastric epithelial cells increased the production of CXCL12 by interacting with p-STAT3 in Helicobacter pylori-associated gastritis, which further aggravated the development of gastritis (9). However, only a few studies have been reported on the function of BHLHE40 in vascular remodeling for now. As demonstrated in our study, TNFα-induced ROS levels and NADPH oxidase activation were attenuated and cell proliferation was reduced in BHLHE40-overexpressed VSMC. In the followed experiments, we found that BHLHE40 blocks VSMC proliferation and oxidative stress by inhibiting TNF-α-induced activation of MAPK signaling pathways.

In line with previous results using E2-treated ligated mice, and our *in vivo* data showed that up-expressed BHLHE40 could significantly reduce carotid artery ligation-induced neointimal formation. Because VSMC proliferation requires the activation of the transcription of several cell cycle promoting genes, we examined the expression of PCNA and cyclin D1 in pcDNA3.1-BHLHE40-transfected injured carotid arteries, beyond that, we also measured the expression of the NADPH oxidase catalytic subunits-NOX1, NOX4, and p47^phox^. Consistent with previous results *in vitro*, and our *in vivo* data showing decreased neointimal thickness via reducing ROS production and VSMC proliferation with localized overexpression of BHLHE40 in injured carotid arteries.

Our results demonstrated for the first time that in TNF-α-stimulated mouse VSMC, E2 diminished VSMC proliferation and oxidative stress via restoring TNF-α-decreased BHLHE40 expression. Furthermore, we explore the possibility that E2 may suppress TNF-α-induced MAPK activity by regulating BHLHE40. In conclusion, our results along with previous studies indicate that E2 exerts the cardiovascular protective effect via-multiple molecular mechanisms, but the accurate mechanism needs further study. This research offers a new molecular explanation for the vasoprotective effect of 17β-estrogen.

## Data Availability Statement

The raw data supporting the conclusions of this article will be made available by the authors, without undue reservation.

## Ethics Statement

The animal study was reviewed and approved by Institutional Animal Care and Use Committee of Hebei Medical University.

## Author Contributions

D-dF and X-hZ conceived and designed the experiments, and wrote the manuscript. D-dF, M-lZ, YM, and XH performed all the experiments. BZ analyzed the data. X-hZ and J-kW engaged in material support for obtained funding and supervised the study. All authors have read and approved the final manuscript.

## Funding

This research was supported by grants from the National Natural Science Foundation of China (Nos. 31871152, 81770285, and 81971328), the Natural Science Foundation of Hebei Province of China (No. H2021206459), and the Postgraduates Innovation Funding Program of Hebei Province (CXZZBS2019122).

## Conflict of Interest

The authors declare that the research was conducted in the absence of any commercial or financial relationships that could be construed as a potential conflict of interest.

## Publisher's Note

All claims expressed in this article are solely those of the authors and do not necessarily represent those of their affiliated organizations, or those of the publisher, the editors and the reviewers. Any product that may be evaluated in this article, or claim that may be made by its manufacturer, is not guaranteed or endorsed by the publisher.
