# Supplementary material for: 17β-Estradiol Inhibits Proliferation and Oxidative Stress in Vascular Smooth Muscle Cells by Upregulating BHLHE40 Expression
Source: Front Cardiovasc Med. 2021 Nov 30;8:768662. doi: 10.3389/fcvm.2021.768662 (PMC8669345; doi:10.3389/fcvm.2021.768662)
Supplement: Supplementary file 1 [file Data_Sheet_1.docx]

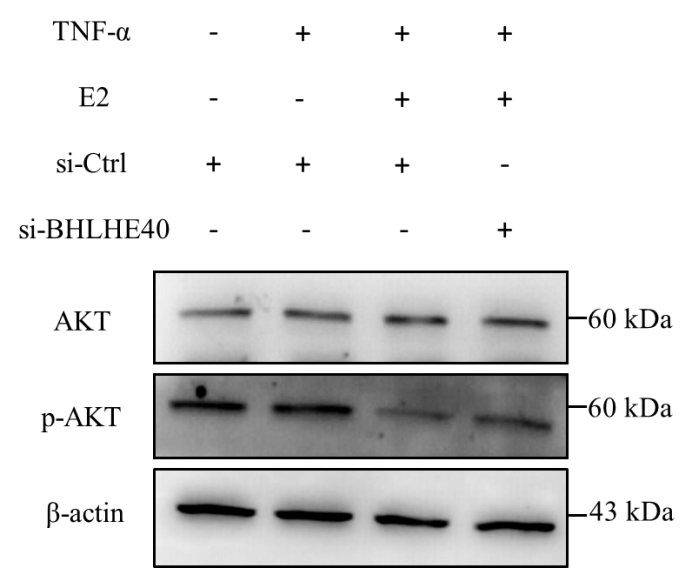


**Supplemental Figure 1**

VSMCs were transfected with si-BHLHE40 or si-Ctrl and then treated with the indicated treatments. The expression of AKT and p-AKT was determined by Western blotting.
